# Prognostic implications of single antiplatelet therapy in individuals developing diabetic foot disease with concurrent peripheral arterial disease

**DOI:** 10.1080/07853890.2026.2653300

**Published:** 2026-04-14

**Authors:** Cheng-Wei Lin, Yu-Yao Huang, Chia-Hung Lin, Chung-Huei Huang, Shih-Yuan Hung, I-Wen Chen, Yi-Chia Chen, Pi-Hua Liu

**Affiliations:** a Department of Endocrinology and Metabolism, Chang Gung Memorial Hospital at Linkou, Taoyuan City, Taiwan; b College of Medicine, Chang Gung University, Taoyuan City, Taiwan; c School of Medicine, National Tsing Hua University, Hsinchu City, Taiwan; d Department of Medical Nutrition Therapy, Chang Gung Memorial Hospital, Taoyuan City, Taiwan; e Graduate Institute of Clinical Medical Sciences, College of Medicine, Chang Gung University, Taoyuan, Taiwan

**Keywords:** Diabetic foot disease, peripheral arterial disease, antiplatelet therapy, lower-extremity amputation, major adverse limb event, major adverse cardiovascular event, survival

## Abstract

**Background:**

Antiplatelet therapy is recommended for secondary prevention in patients with diabetes and peripheral arterial disease (PAD), particularly after a diabetic foot event. Nevertheless, the relative effectiveness of aspirin, clopidogrel, and cilostazol for long-term outcomes remains uncertain.

**Methods:**

Using the Taiwan Health and Welfare Data Center database, we identified 2,597 adults with type 2 diabetes who experienced their first diabetic foot disease (DFD) event with concurrent PAD between 2016 and 2019 and subsequently received a single antiplatelet agent for secondary prevention after stabilization. Outcomes included major lower extremity amputation (LEA), major adverse limb events (MALE), major adverse cardiovascular events (MACE), and all-cause mortality. Inverse probability of treatment weighting (IPTW)-adjusted Cox proportional hazards and Fine–Gray competing risk models were used to estimate hazard ratios (HRs) and subdistribution HRs (sHRs) with 95% confidence intervals (CIs).

**Results:**

Baseline characteristics indicated higher comorbidity burdens in the clopidogrel and cilostazol groups. After adjustment, the risks of LEA and MACE did not differ significantly among treatment groups. Compared with aspirin, cilostazol was associated with a higher risk of MALE (sHR 1.45 [95% CI 1.15–1.84]), whereas clopidogrel showed a nonsignificant trend (sHR 1.31 [95% CI 0.99–1.72]). Both cilostazol (HR 1.21 [95% CI 1.06–1.39]) and clopidogrel (HR 1.25 [95% CI 1.07–1.46]) were associated with higher all-cause mortality. Exploratory subgroup analyses showed no significant mortality differences among dialysis patients, and sensitivity analyses yielded consistent results.

**Conclusions:**

Among diabetic patients with PAD and a history of a foot event, aspirin was associated with comparable limb and cardiovascular outcomes, and potentially favorable survival, compared with clopidogrel or cilostazol. These observational findings suggest aspirin is a practical antiplatelet option for secondary prevention in this high-risk population, though further precise trials are needed to verify.

## Graphical Abstract

**Figure:**
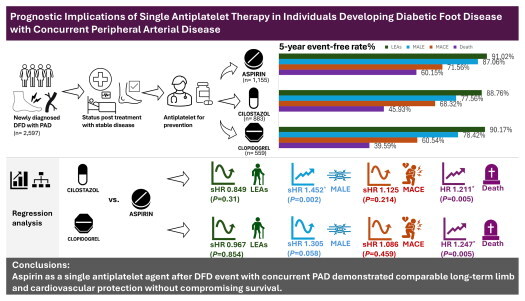

## Introduction

Diabetes dramatically elevates the risk of vascular events, leaving patients two to four times more vulnerable to major cardiovascular complications than individuals without diabetes [1]. This heightened risk is attributable to a prothrombotic environment in diabetes, which results from multiple pathological processes, including hyperglycemia and chronic inflammation, that damage the vascular endothelium and increase platelet reactivity [2]. While lifestyle changes and optimal glycemic, blood pressure, and lipid management are the cornerstones of diabetes control, antiplatelet therapy is also crucial in preventing cardiovascular complications [3]. For individuals with diabetes but no prior cardiovascular events, the use of antiplatelet therapy for primary prevention requires careful consideration [3] due to its controversial net benefit [1,4,5]. Conversely, the American Diabetes Association strongly recommends antiplatelet therapy for secondary prevention in diabetic patients with established cardiovascular or cerebrovascular disease [3]. However, this guidance aimed to reduce cardiovascular morbidity and mortality, without addressing lower limb prognosis.

Peripheral arterial disease (PAD) is also a manifestation of systemic atherosclerosis that leads to high cardiovascular morbidity and mortality [6]. People with diabetes are at higher risk of developing PAD. Despite its prevalence, PAD often remains underdiagnosed or is detected late in patients with diabetes. This is because neuropathy can mask the classic symptom of intermittent claudication, and arterial calcification impairs the accuracy of the ankle-brachial pressure index due to falsely elevated readings [7]. Consequently, current clinical guidelines recommend utilizing alternative diagnostic strategies, such as the toe-brachial pressure index or Doppler waveform analysis, which are less susceptible to calcification artifacts and provide a more accurate assessment of perfusion in this population [8,9]. Indeed, most diabetics with PAD are asymptomatic and may only become aware of their disease after developing a foot event, such as a foot ulcer or foot infection. For these patients with diabetes and PAD who develop diabetic foot disease (DFD), long-term single antiplatelet therapy is typically recommended for secondary prevention in stable disease following successful limb salvage [10]. In contrast, while short-term dual antiplatelet therapy (typically 1–6 months) may be considered following revascularization to reduce the risk of limb events (Class IIb recommendation) [8], prolonged use has not demonstrated added benefit in stable PAD and instead increases bleeding risk [10]. More intensive antithrombotic strategies have been considered in high-risk cases. Low-dose rivaroxaban in combination with aspirin is considered for PAD patients at high risk of limb loss or with high-risk cardiovascular comorbidities, absent a high bleeding risk [8,9]. However, diabetes, particularly when poorly controlled, can be associated with an increased risk of bleeding due to its impact on coagulation and vessel health [11–13]; therefore, dual antithrombotic therapy should be initiated with caution.

Regarding the antiplatelet agents themselves, aspirin remains the most widely used medication for secondary prevention in reducing cardiovascular events for patients with diabetes [3]. Clopidogrel, a P2Y12 receptor antagonist, demonstrated slightly greater efficacy than aspirin in the CAPRIE trial, with a significantly lower rate of ischemic events (23.8% relative risk reduction vs. aspirin) observed in the PAD subgroup [14]. However, this result was not specific to diabetes and lacked limb outcome analysis. Another antiplatelet agent, cilostazol, a phosphodiesterase-3 inhibitor, is approved for the treatment of intermittent claudication to improve walking distance. Its mechanism involves not only platelet inhibition but also vasodilation, achieved by increasing cyclic adenosine monophosphate (cAMP) levels in vascular smooth muscle cells, thereby increasing blood flow [15]. However, evidence of long-term benefit on cardiovascular outcomes or limb preservation is lacking, and the result is not specific to diabetes either [16].

This study aimed to better understand the long-term prognosis, including limb, cardiovascular, and survival, among patients with type 2 diabetes and PAD who had experienced a foot event with subsequent stable disease from 2016 to 2019. To address the insufficiency of real-world data, we specifically compared patients treated with single antiplatelet therapy, including aspirin, clopidogrel, and cilostazol, to determine which agent is associated with the most favorable outcomes in terms of preventing future limb complications and cardiovascular events in this population.

## Materials and methods

### Study population and source

The present study utilized data from the Health and Welfare Data Science Center, Ministry of Health and Welfare (HWDC, MOHW) spanning 2011 to 2021, with data analysis conducted from January 2024 to September 2025. The Taiwanese government established its National Health Insurance (NHI) system in 1995, providing healthcare services to nearly 99% of the Taiwanese population with the consequent development of the National Health Insurance Research Database (NHIRD), a comprehensive population-level data source that supports real-world research and informs healthcare policy decisions [17–19]. All data access and analyses were conducted in strict compliance with the Ministry of Health and Welfare’s standard protocols for privacy and security [17]. Patients with type 2 diabetes mellitus were identified by diagnostic codes indicating at least one diabetes-related hospitalization according to the ICD-10 diagnostic code of E11 or had been treated in diabetes-related outpatient services on ≥ three occasions. We focused exclusively on type 2 diabetes to maintain a homogeneous study cohort, given the distinct pathophysiological mechanisms of type 1 diabetes and its lower prevalence in this population. Patients with a foot disease, including foot ulcers, infections, or gangrene, and concurrent PAD were identified using the ICD-10 codes detailed in the Supplementary Appendix 1. The diagnosis of PAD in this study was also established exclusively *via* ICD-10 diagnostic codes indicating peripheral angiopathy. To avoid overestimating DFD rates by including patients with multiple foot complications, only the first occurrence of any identified DFD was designated as the index date. We identified adults (aged 18 and older) with new-onset DFD between 2016 and 2019, ensuring no prior occurrence in the preceding five years. To ensure a stable baseline cohort for follow-up, patients were excluded if they experienced a major lower extremity amputation, a major adverse cardiac event (MACE), or death within 90 days following the index date. The Chang Gung Medical Foundation Institutional Review Board approved this study and waived the need for participant consent. The study’s reporting conforms to the STROBE guideline [20]. We conducted our study in accordance with the Helsinki Declaration of 1975 as revised in 2024.

### Antiplatelet therapy, clinical characteristics, and concomitant medications

Following the intention-to-treat principle, the final study cohort was classified into three mutually exclusive groups based on their antiplatelet therapy, which included aspirin, cilostazol, or clopidogrel. A patient was assigned to a group if they received at least one prescription for the corresponding drug lasting 28 days or more within the first three months after the index foot event. To minimize confounding from drug interactions, we excluded patients who were exposed to more than one type of antiplatelet agent or any anticoagulation therapy during these three months. Because we utilized an administrative claims database, the daily dosages of aspirin were unspecified. However, in Taiwan’s clinical practice, the standard prescribed dosage for secondary cardiovascular prevention is low-dose aspirin (typically 100 mg daily). Covariates included age, gender, baseline comorbidities, and concomitant medications. Comorbidities, including hypertension, dyslipidemia, coronary heart disease (CHD), cerebral vascular accident (CVA), heart failure, renal disease, diabetic retinopathy or neuropathy, were defined by at least one inpatient or two outpatient diagnoses before the index date. Concomitant medications such as anti-diabetes drugs, antihypertensive agents, and statins were identified using the same ≥28 days prescription rule during the three-month post-index period.

### Definition of subgroups of kidney diseases

Given that kidney disease is a frequent comorbidity in this population that independently alters bleeding risk, thrombotic potential, and the pharmacodynamic response to antiplatelet agents [21,22], we performed subgroup analysis stratified by renal function. Patients with varying degrees of kidney disease were identified using diagnostic codes (Supplementary Appendix 1). Those requiring long-term dialysis were classified as having end-stage renal disease (ESRD), while the remaining patients were categorized as having diabetic kidney disease (DKD). The demand for dialysis was identified as undergoing dialysis for at least three successive months, including hemodialysis (NHI procedure codes: 58001, 58019, 58020, 58021, 58022, 58023, 58024, 58025, and 58029 C), peritoneal dialysis (58002 C, 58009 A, 58009B, 58010 A, 58010B, 58011 A, 58011AB, 58011B, 58011 C, 58012 A, 58012B, 58017B, 58017, 58026, and 58028 C), and other dialysis (58018 C, 58027 C, and 58030B) [23].

### Outcomes evaluation

**Figure 1.**
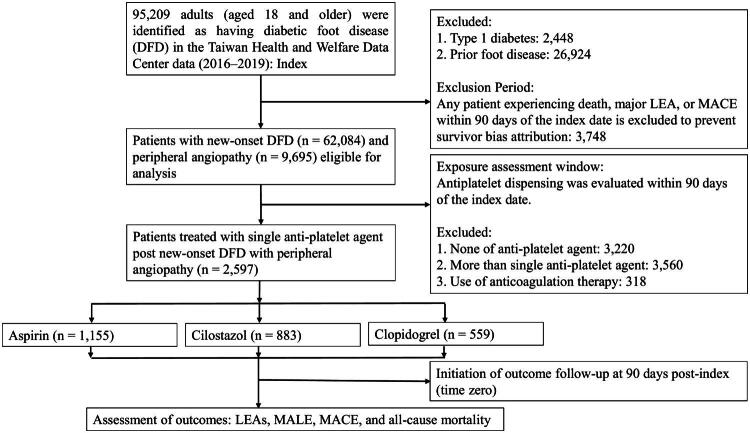
Flowchart of patient selection. Individuals with type 2 diabetes who received a single antiplatelet agent for secondary prevention after a new event of diabetic foot disease (DFD) with peripheral angiopathy were included in the analysis after applying exclusion criteria.

The primary endpoints for analysis in this study included lower-extremity amputation (LEA), major adverse limb event (MALE), major adverse cardiac event (MACE), and all-cause mortality. Lower-extremity amputations were identified by diagnostic codes or procedure codes (Supplementary Appendix 1) for minor LEA (below the ankle) and major LEA (above the ankle) respectively. MALE was defined as a composite outcome including a major lower-extremity amputation or a repeat endovascular or surgical bypass procedure [24]. The endovascular treatments were defined by the procedure codes for percutaneous transluminal angioplasty (PTAs), either simple PTA (33074B) or complicated PTA (33115B). The bypass graft surgery was defined by code of 69004B. These specific procedure codes of NHIRD are provided in Supplementary Appendix 2. The MACE was defined as a composite outcome of nonfatal myocardial infarction, nonfatal stroke, unstable angina, hospitalization for heart failure, and cardiovascular death. By initiating outcome follow-up at 90 days post-index (time zero) and excluding the initial 90-day exposure classification window from the at-risk follow-up time, the structural conditions for immortal time bias were eliminated (Figure 1). All patients were followed until the end of 2021 to determine the occurrence of any study endpoints.

### Statistical analysis

Baseline demographic and clinical characteristics across the three antiplatelet therapy groups (aspirin, clopidogrel, and cilostazol) were summarized as frequencies and percentages for categorical variables and as means with standard deviations (SDs) for continuous variables. Between-group differences were assessed using Pearson’s chi-square test for categorical variables and one-way analysis of variance (ANOVA) for continuous variables. Time-to-event analyses were performed to evaluate clinical outcomes. Inverse probability of treatment weighting (IPTW) based on propensity scores was applied to construct a weighted pseudo-population. Weighted Cox proportional hazards regression models were used to estimate hazard ratios (HRs) and 95% confidence intervals (CIs) for all-cause mortality. For nonfatal outcomes, including MALE, LEA, and MACE, cumulative incidence functions were compared across groups using the weighted Fine and Gray subdistribution hazard model, with death considered as a competing risk. Subgroup analyses stratified by renal status (non-DKD, DKD, and ESRD requiring dialysis) were considered exploratory. To assess whether the treatment effects differed significantly across renal strata, formal interaction testing was performed by including cross-product interaction terms in the regression models. Interaction *P* values were reported to contextualize these exploratory findings. Sensitivity analyses included (1) repeating the analyses using an unweighted multivariable Cox model, (2) excluding patients who died within the first 90 days after the index event, and (3) performing subgroup analyses stratified by age, sex, and cardiovascular comorbidities. All statistical tests were two-sided, and a *P* value <0.05 was considered statistically significant. Analyses were performed using SAS version 9.4 (SAS Institute, Cary, NC, USA).

## Results

### The inclusion of study participants

Between January 2016 and December 2019, a total of 95,209 adults (≥ 18 years) were identified in the Taiwan Health and Welfare Data Center as having diabetic foot disease (DFD). There were 62,084 patients with newly diagnosed DFD after excluding 2,448 individuals with type 1 diabetes, 26,924 with any prior foot disease, and 3,748 who sustained a major lower-extremity amputation (LEA), major adverse cardiac event (MACE), or death within 90 days of the index date. Among these, 9,695 patients had diagnostic codes consistent with peripheral angiopathy and were considered for inclusion in the present study. We further excluded 3,220 patients who did not receive any antiplatelet therapy, 3,560 who were prescribed more than one antiplatelet agent, and 318 who received concurrent anticoagulation. The final study cohort comprised 2,597 patients with type 2 diabetes and DFD with peripheral arterial disease (PAD) who received a single antiplatelet agent for secondary prevention. Of these, 1,155 (44.5%) received aspirin, 883 (34.0%) received cilostazol, and 559 (21.5%) received clopidogrel (Figure 1).

### Characteristics of patients with DFD and PAD across antiplatelet therapies

Baseline demographic and clinical characteristics of the three treatment groups are summarized in Table 1. The mean age differed significantly among groups (69.31 ± 11.3 years in the aspirin group, 72.48 ± 12.27 years in the cilostazol group, and 73.17 ± 10.91 years in the clopidogrel group) (*p* < 0.001). The proportion of male patients was similar across all groups (approximately 55%, *p* = 0.205).

**Table 1.** Clinical characteristics of individuals with diabetic foot disease and peripheral arterial disease across different antiplatelet therapies.

	Aspirin (*n* = 1,155)	Cilostazol (*n* = 883)	Clopidogrel (*n* = 559)	*P* value	Pre-IPTW SMD* (Cilostazol/Clopidogrel)	Post-IPTW SMD* (Cilostazol/Clopidogrel)
Characteristic						
Age	69.31 ± 11.30	72.48 ± 12.27	73.17 ± 10.91	<0.001	−0.269/−0.348	−0.052/−0.042
Gender				0.205	0.079/0.025	0.021/0.022
Male	655 (56.71%)	466 (52.77%)	310 (55.46%)			
Female	500 (43.29%)	417 (47.23%)	249 (44.54%)			
Comorbidities						
Hypertension	881 (76.28%)	636 (72.03%)	458 (81.93%)	<0.001	0.097/−0.139	−0.007/−0.028
Dyslipidemia	606 (52.47%)	426 (48.24%)	255 (45.62%)	0.018	0.085/0.137	−0.006/−0.009
Diabetic retinopathy	158 (13.68%)	141 (15.97%)	96 (17.17%)	0.125	−0.064/−0.097	0.013/0.043
Diabetic neuropathy	185 (16.02%)	169 (19.14%)	112 (20.04%)	0.066	−0.082/−0.105	−0.007/0.008
Coronary heart disease	445 (38.53%)	191 (21.63%)	272 (48.66%)	<0.001	0.375/−0.205	−0.005/−0.023
Cerebrovascular accident	286 (24.76%)	152 (17.21%)	195 (34.88%)	<0.001	0.186/−0.223	−0.041/−0.012
Heart failure	94 (8.14%)	58 (6.57%)	87 (15.56%)	<0.001	0.060/−0.231	0.004/−0.029
Dialysis	150 (12.99%)	200 (22.65%)	153 (27.37%)	<0.001	0.254/0.364	0.010/0.001

*IPTW SMD: Inverse probability of treatment weighting standardized mean difference.

Regarding comorbidities, the prevalence of hypertension was highest among clopidogrel users (81.93%) and lowest among cilostazol users (72.03%; *p* < 0.001). Dyslipidemia was noted in 52.47% of those using aspirin, 48.24% of those using cilostazol, and 45.62% in the clopidogrel group (*p* = 0.018). Macrovascular diseases, including coronary heart disease (CHD) and cerebrovascular accident (CVA), were most common among clopidogrel users (CHD 48.66%, CVA 34.88%), compared with the aspirin (38.53%, 24.76%) and cilostazol (21.63%, 17.21%) users (both *p* < 0.001). Heart failure was also more prevalent in the clopidogrel group (15.56%) than among aspirin (8.14%) or cilostazol (6.57%) groups (*p* < 0.001). Differences in renal status were notable. The proportion of patients requiring dialysis was 27.37% in the clopidogrel users and 22.65% in the cilostazol users, compared with 12.99% among aspirin users (*p* < 0.001).

Patterns of background medical treatment also varied by antiplatelet agent (Table 2). Aspirin users had the highest prevalence of metformin (56.54%) and sulfonylurea (41.39%), whereas cilostazol users were more likely to receive glinides (13.7%) (all *p* < 0.001). The use of sodium-glucose co-transporter 2 inhibitors (SGLT2-i) was 7.19% in the aspirin group, 4.42% in the cilostazol group, and 3.94% in the clopidogrel group (*p* = 0.004). The use of injection therapies was comparable across groups, with approximately 1% using glucagon-like peptide 1 (GLP-1) analog and 33% using insulin (*p* = 0.21 and *p* = 0.42, respectively). Prescription patterns for antihypertensive medications also varied significantly, with higher use of renin-angiotensin-aldosterone system (RAAS) blockade and calcium-channel blockers in the aspirin group and more frequent β-blocker use in the clopidogrel group (all *p* < 0.001). Statins were prescribed to 53.94% of aspirin users, versus 39.3% and 49.55% of cilostazol and clopidogrel users, respectively (*p* < 0.001).

**Table 2.** Diabetes-related medications of participants across different antiplatelet therapies.

	Aspirin (*n* = 1,155)	Cilostazol (*n* = 883)	Clopidogrel (*n* = 559)	*P* value	Pre- IPTW SMD* (Cilostazol/Clopidogrel)	Post- IPTW SMD* (Cilostazol/Clopidogrel)
Anti-Diabetes drugs						
Sulfonylurea	478 (41.39%)	292 (33.07%)	155 (27.73%)	<0.001	0.173/0.290	0.075/0.154
Metformin	653 (56.54%)	390 (44.17%)	186 (33.27%)	<0.001	0.249/0.481	0.112/0.249
Acarbose	117 (10.13%)	69 (7.81%)	52 (9.3%)	0.198	0.081/0.028	0.035/−0.007
Glinide	104 (9%)	121 (13.7%)	76 (13.6%)	0.001	−0.149/−0.146	−0.079/−0.021
Pioglitazone	121 (10.48%)	75 (8.49%)	28 (5.01%)	0.001	0.068/0.206	0.048/0.182
DPP4-i	491 (42.51%)	370 (41.9%)	259 (46.33%)	0.217	0.012/−0.077	−0.029/−0.088
SGLT2-i	83 (7.19%)	39 (4.42%)	22 (3.94%)	0.004	0.119/0.142	−0.012/−0.002
Insulin	377 (32.64%)	293 (33.18%)	200 (35.78%)	0.422	−0.011/−0.066	0.022/0.006
GLP-1 analog	19 (1.65%)	7 (0.79%)	6 (1.07%)	0.209	0.078/0.050	−0.011/0.021
Antihypertensive agents						
RAAS blockade	662 (57.32%)	373 (42.24%)	291 (52.06%)	<0.001	0.305/0.106	−0.001/0.025
Calcium channel blocker	640 (55.41%)	406 (45.98%)	273 (48.84%)	<0.001	0.189/0.132	0.079/0.095
β-blocker	447 (38.7%)	236 (26.73%)	230 (41.14%)	<0.001	0.257/−0.050	−0.010/−0.037
Statin	623 (53.94%)	347 (39.3%)	277 (49.55%)	<0.001	0.297/0.088	−0.006/−0.002

*IPTW SMD: Inverse probability of treatment weighting standardized mean difference.

The distribution of antiplatelet agents in our cohort likely reflects specific clinical decision-making patterns. The higher utilization of cilostazol compared to clopidogrel may be attributed to its specific indication for improving limb perfusion [15], given the high burden of PAD in this population. In contrast, patients prescribed clopidogrel had a significantly higher burden of macrovascular comorbidities, including coronary heart disease and cerebrovascular accident. This likely reflects confounding by indication, where clinicians preferentially selected clopidogrel for patients with established atherosclerotic cardiovascular disease, consistent with evidence supporting its efficacy in secondary prevention [25]. To mitigate this selection bias, we utilized IPTW to balance the baseline risk profiles across groups, ensuring that the observed outcomes were not driven by these pre-existing disparities (Tables 1 and 2). Before weighting, standardized mean differences (SMDs) revealed notable imbalances across treatment groups, particularly in age, cardiovascular comorbidities, kidney disease, and use of metformin and sulfonylureas. After applying inverse probability of treatment weighting (IPTW), post-weighting SMDs were substantially reduced, indicating adequate covariate balance for subsequent outcome analyses.

### Outcomes analysis between different antiplatelet therapies

**Figure 2.**
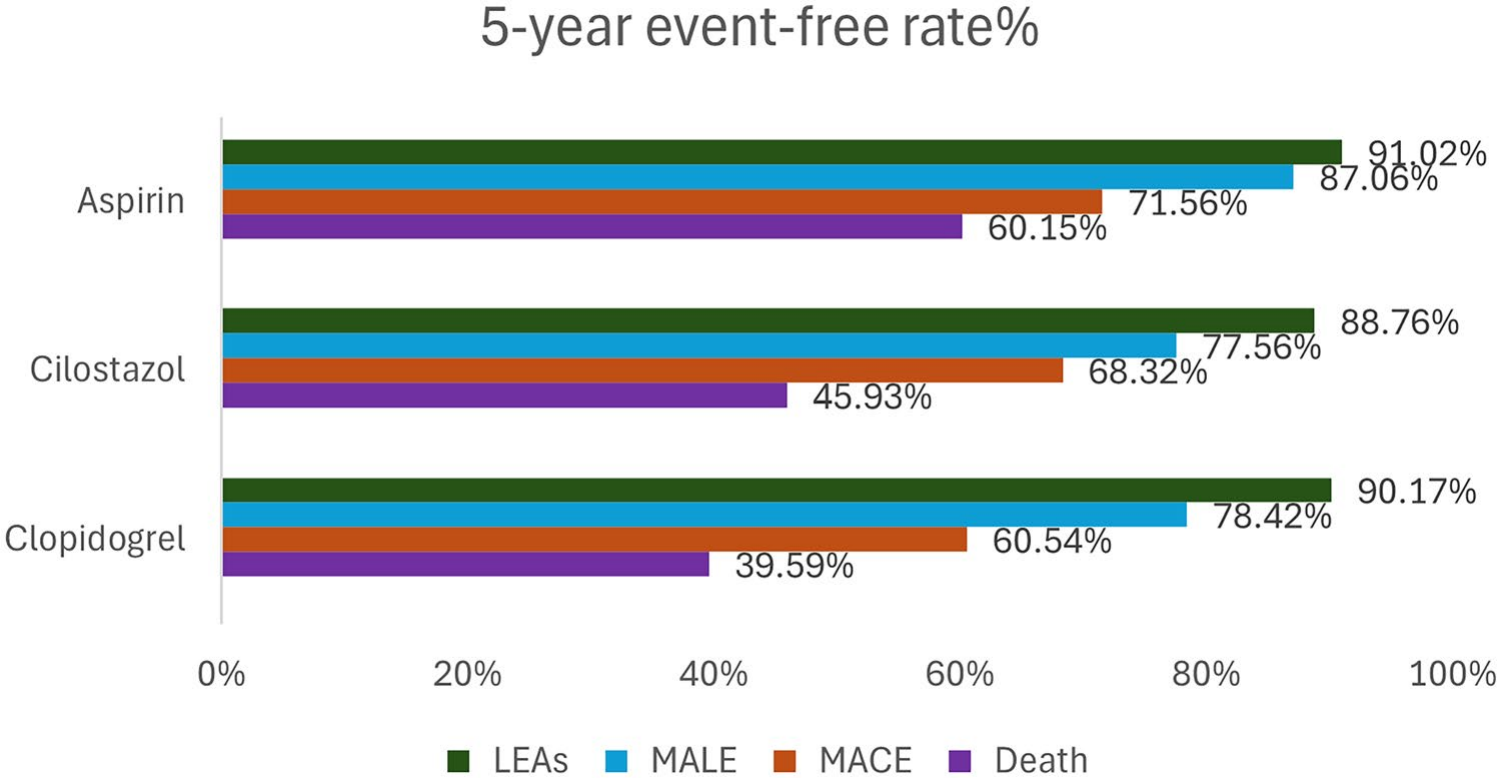
Five-year event-free rates (%) in patients with diabetic foot disease and peripheral angiopathy receiving single antiplatelet therapy (aspirin, cilostazol, or clopidogrel). The 5-year event-free rate for LEA was comparable among the aspirin (91.02%), cilostazol (88.76%), and clopidogrel (90.17%) groups. For MALE, the event-free rate was highest in the aspirin group (87.06%) compared to the cilostazol (77.56%) and clopidogrel (78.42%) groups. The aspirin users also had the highest event-free rates in MACE and all-cause mortality (71.56% and 60.15%, respectively) compared to the others. (LEA: lower-extremity amputation; MALE: major adverse limb event; MACE: major adverse cardiac event.)

Clinical outcomes among patients with DFD and PAD were evaluated across four endpoints, including LEAs, MALE, MACE and all-cause mortality (Figure 2). During follow-up, the median follow-up duration was 3.00 years (IQR 2.02–4.28) for the all-cause mortality analysis; 2.82 years (IQR 1.70–4.18) for LEA, 2.72 years (IQR 1.50–4.08) for MALE, and 2.64 years (IQR 1.43–3.97) for MACE. The 5-year event-free rate of LEA was comparable among the groups, with rates of 91.02% for aspirin, 88.76% for cilostazol, and 90.17% for clopidogrel. For MALE, the corresponding 5-year event-free rates were 87.06%, 77.56% and 78.42%, respectively. Regarding MACE and all-cause mortality, the aspirin group had the highest 5-year event-free rates (71.56% and 60.15%, respectively), followed by cilostazol (68.32% and 45.93%) and clopidogrel (60.54% and 39.59%).

**Figure 3.**
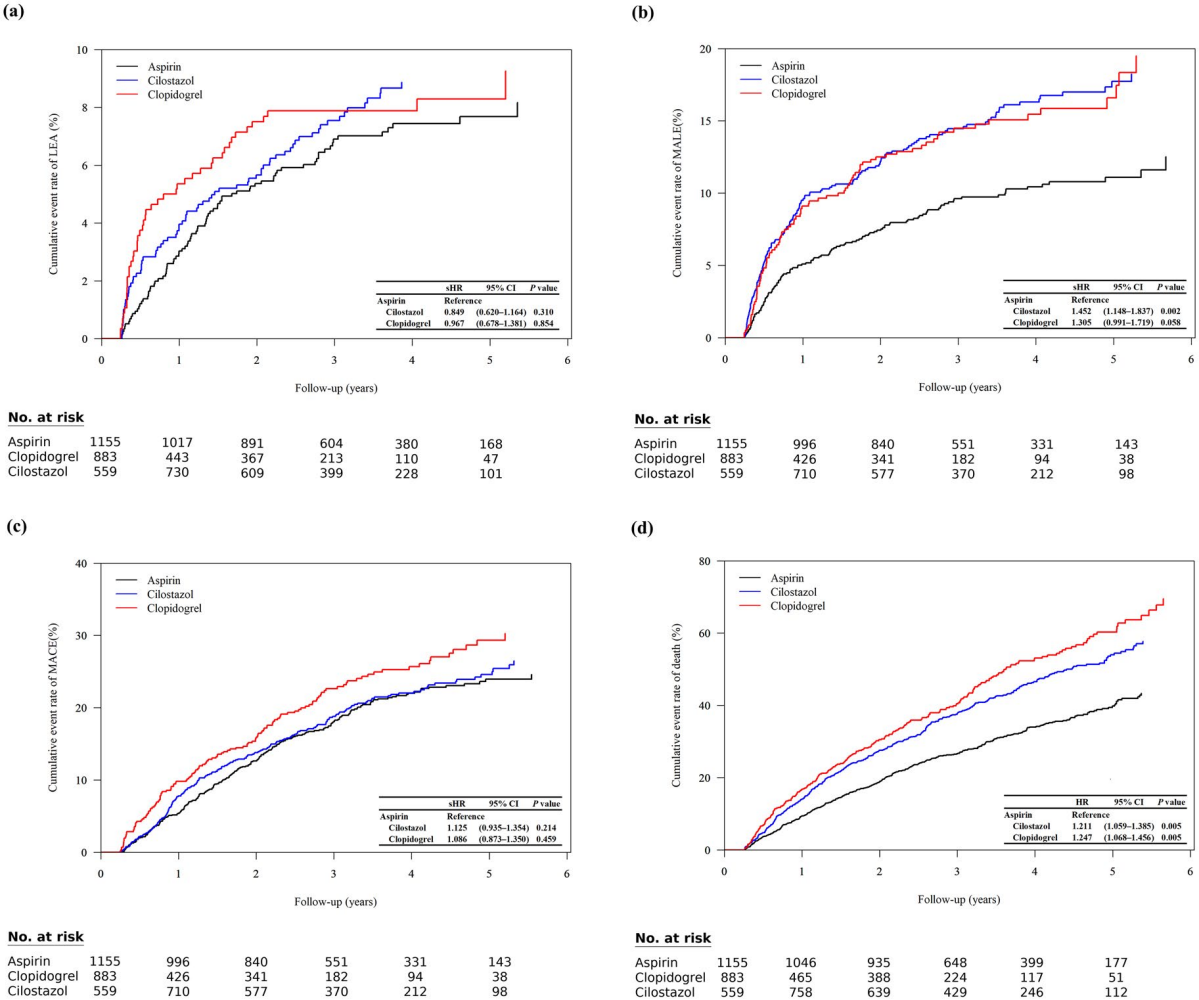
Cumulative incidence functions and survival curves for primary outcomes. (a) Lower extremity amputation (LEA): The risk did not differ significantly when comparing either cilostazol or clopidogrel to aspirin. (b) Major adverse limb events (MALE): The risk was significantly higher with cilostazol (sHR 1.452, 95% CI 1.148–1.837) and non-significantly higher with clopidogrel (sHR 1.305, 95% CI 0.991–1.719) compared to aspirin. (c) Major adverse cardiovascular events (MACE): No significant differences were observed. (d) All-Cause Mortality: Both cilostazol (HR 1.211, 95% CI 1.059–1.385) and clopidogrel (HR 1.247, 95% CI 1.068–1.456) were associated with a significantly higher risk of all-cause mortality compared to aspirin. Curves are stratified by antiplatelet therapy.

After multivariable adjustment using IPTW, including baseline demographics (age and gender), comorbidities (hypertension, dyslipidemia, CHD, CVA, and heart failure), diabetes complications, and medications (RAAS blockade, β-blocker, statin, SGLT2-i, and GLP-1 analog), neither cilostazol nor clopidogrel significantly altered the risk of LEAs when compared with aspirin (sub-distribution hazard ratio [sHR] 0.849, 95% CI 0.620–1.164, *p* = 0.31 for cilostazol; sHR 0.967, 95% CI 0.678–1.381, *p* = 0.854 for clopidogrel) (Figure 3). In contrast, the risk of the composite outcome MALE differed significantly among treatments. Cilostazol was associated with a 45% higher risk of MALE relative to aspirin (sHR 1.452, 95% CI 1.148–1.837, *p* = 0.002), while clopidogrel showed a non-significant finding (sHR 1.305, 95% CI 0.991–1.719, *p* = 0.058). When deconstructing the composite MALE endpoint, the events were predominantly driven by repeat revascularization procedures rather than major amputations. Among the 349 total MALE events observed during follow-up, 274 (78.5%) were revascularizations, while 75 (21.5%) were major LEAs. Furthermore, a sensitivity analysis of cilostazol, focusing exclusively on the hard endpoint of major LEA, showed no significant difference compared with aspirin (sHR 1.287, 95% CI 0.739–2.241, *p* = 0.372). Conversely, the association between cilostazol and repeat revascularization remained significant (sHR 1.473, 95% CI 1.135–1.910, *p* = 0.004). For MACE, no significant differences were observed among the three agents (cilostazol sHR 1.125, 95% CI 0.935–1.354, *p* = 0.214; clopidogrel sHR 1.086, 95% CI 0.873–1.350, *p* = 0.459). During follow-up, 1,081 deaths occurred. In fully adjusted Cox models, both cilostazol and clopidogrel were associated with higher all-cause mortality compared with aspirin. The hazard ratio was 1.211 for cilostazol (95% CI 1.059–1.385, *p* = 0.005) and 1.247 for clopidogrel (95% CI 1.068–1.456, *p* = 0.005). To quantify the potential magnitude of this unmeasured confounding, we calculated E-values for our all-cause mortality findings. For the association between clopidogrel and increased mortality (HR 1.247), the E-value was 1.802 (lower confidence limit: 1.337). For cilostazol (HR 1.211), the E-value was 1.717 (lower confidence limit: 1.309). This indicates that an unmeasured confounder would need to be associated with the choice of antiplatelet therapy.

### Subgroup analysis across renal status

**Figure 4.**
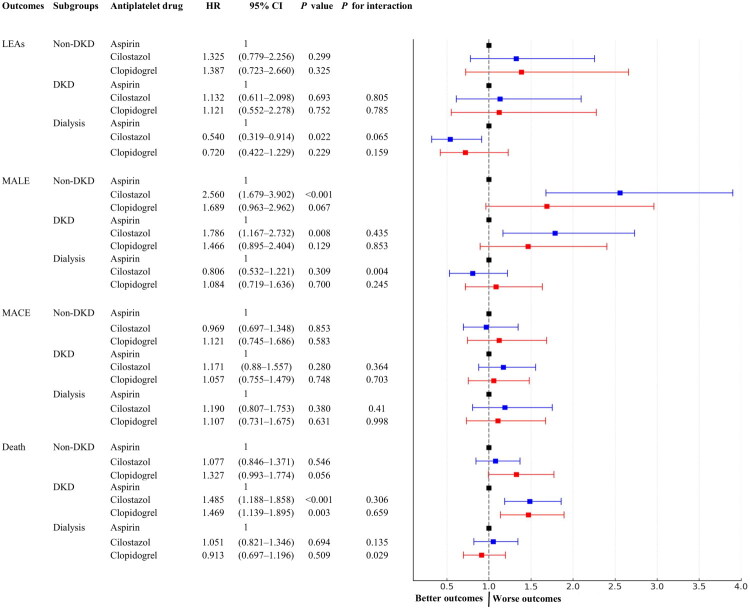
Forest plot of hazard ratios for primary outcomes among patients with diabetic foot disease and peripheral angiopathy, stratified by renal status. The risk of LEA did not differ between antiplatelet therapies in the non-DKD and DKD subgroups; however, among patients on dialysis, cilostazol was associated with a reduced risk of LEA (sHR 0.540, 95% CI, 0.319–0.914). For MALE, cilostazol was associated with a higher risk than aspirin in both the non-DKD (sHR, 2.56; 95% CI, 1.679–3.902) and DKD subgroups (sHR, 1.786; 95% CI, 1.167–2.732. The risk of MACE did not differ in any stratum. For all-cause mortality, both cilostazol (HR 1.485; 95% CI, 1.188–1.858) and clopidogrel (HR 1.469; 95% CI, 1.139–1.895) were associated with a higher risk compared to aspirin in the DKD subgroup.

Exploratory subgroup analyses were conducted based on baseline renal status: patients without diabetic kidney disease (non-DKD), patients with DKD not requiring dialysis, and those receiving maintenance dialysis (Figure 4).

For LEA, although cilostazol appeared to be associated with a lower LEA risk in the dialysis stratum (sHR 0.54, 95% CI 0.319–0.914, *p* = 0.022), the interaction test comparing dialysis versus non-DKD did not reach conventional significance (interaction *p* = 0.065). Therefore, this finding is interpreted as strictly exploratory and is not considered definitive evidence of a subgroup benefit.

For MALE, we observed significant heterogeneity in the cilostazol-renal status (dialysis vs. non-DKD, interaction *p* = 0.004); however, the regression analysis showed no significant risk in the dialysis subgroup itself (sHR 0.806, 95% CI 0.532–1.221, *p* = 0.309). Conversely, while an elevated MALE risk was noted for cilostazol within the DKD subgroup (sHR 1.786, 95% CI 1.167–2.732, *p* = 0.008), the interaction test comparing DKD versus non-DKD was not significant (interaction *p* = 0.435).

The risk of MACE did not significantly differ among the three antiplatelet groups across any renal strata, and no significant interactions were noted. Compared with aspirin, the sHRs for cilostazol and clopidogrel were 0.969 and 1.121 in the non-DKD group, 1.171 and 1.057 in the DKD group, and 1.190 and 1.107 in the dialysis group, respectively.

For all-cause mortality, a significant interaction was observed for clopidogrel (dialysis vs. non-DKD, interaction *p* = 0.029), but the mortality risk was not statistically significant within the dialysis subgroup itself (HR 0.913, 95% CI 0.697–1.196, *p* = 0.509). Finally, although both cilostazol (HR 1.485, 95% CI 1.188–1.858, *p* < 0.001) and clopidogrel (HR 1.469, 95% CI 1.139–1.895, *p* = 0.003) showed higher mortality compared with aspirin specifically within the DKD subgroup, no significant interaction was noted for this specific stratum. Because this observation is based on a smaller sample size and wide confidence intervals, it necessitates cautious interpretation.

## Discussion

This cohort study provides important insights into the comparative effectiveness of aspirin, clopidogrel, and cilostazol in patients with diabetes and PAD following a DFD event. Overall, we found no significant difference in the incidence of lower extremity amputations among the three single antiplatelet therapies. However, cilostazol was associated with a significantly higher risk of MALE (a composite of major amputation and revascularization) compared to aspirin, while clopidogrel showed a non-significant trend toward an increased risk. Furthermore, both cilostazol and clopidogrel were associated with higher all-cause mortality than aspirin, whereas the risk of MACE was comparable across all three groups.

Our findings initially indicated a higher incidence of MALE with cilostazol. However, deconstructing this composite endpoint revealed that the events were almost exclusively driven by repeat revascularizations rather than major amputations. This result might be due to the potential for suboptimal adherence; the recommended twice-daily dosing of cilostazol contrasts with the simpler once-daily regimen of aspirin, and this increased pill burden may lead to poorer compliance and reduced therapeutic efficacy [26]. Nevertheless, the increased revascularization rate in the cilostazol cohort must be interpreted with caution. Because cilostazol is primarily indicated for the symptomatic relief of intermittent claudication to improve walking distance, patients prescribed this agent likely had more pronounced baseline ischemic symptoms. We acknowledge that, due to limitations of our administrative database, we could not distinguish between urgent revascularizations driven by acute ischemia and elective procedures in which cilostazol might have been used for perioperative optimization [27]. Consequently, these patients may have been subject to closer vascular surveillance. Therefore, the higher MALE rate may reflect surveillance bias and a greater intensity of clinical care rather than the drug’s inferiority in preventing acute limb ischemia.

In the comparison of clopidogrel and aspirin, our study found no significant advantage for clopidogrel in preventing MALE or MACE. This contrasts with prior research, including the CAPRIE trial [14] and a comprehensive network meta-analysis [28], which demonstrated clopidogrel’s superiority over aspirin in reducing cardiovascular events. However, those studies were not restricted to a diabetic cohort. Additionally, the CAPRIE subgroup analysis revealed a significant benefit for clopidogrel solely in the PAD subgroup (relative risk reduction [RRR]: 8.7%, *p* = 0.045), but not in patients with stroke (RRR: 7.3%, *p* = 0.26) or myocardial infarction (RRR: −3.7%, *p* = 0.66). It is also notable that the 325 mg daily aspirin dosage used in CAPRIE significantly exceeds the currently recommended 75–100 mg daily dose; this higher dosage was associated with increased bleeding severity, which may have impacted adherence and outcomes.

Moreover, the finding that both clopidogrel and cilostazol were associated with higher mortality than aspirin is unexpected and requires cautious interpretation. Given clopidogrel’s established efficacy in populations with major vascular disease, this mortality difference is unlikely to represent true biological inferiority. Because our administrative dataset lacks the granularity to assess precise frailty scores, functional status, or the complete anatomical severity of atherosclerotic burden, this survival disparity is plausibly driven by residual confounding by indication. Consequently, these findings represent associations rather than evidence of a causal survival benefit associated with aspirin. Despite being unexpected, our results align with certain existing literature. A meta-analysis of patients with coronary artery disease observed no mortality difference between clopidogrel and aspirin [29], while a real-world study in an Asian population reported higher mortality with clopidogrel used for stroke prevention [30]. Regarding cilostazol, although it is effective for intermittent claudication and secondary stroke prevention, a clear survival benefit has not been established [31].

In exploratory subgroup analyses stratified by renal status, the previously noted variations in MALE and survival across the three antiplatelet therapies were no longer statistically significant among patients with ESRD on dialysis. The similar event rates suggest that no single agent provides a clear outcome advantage in this high-risk subgroup. A likely explanation is that the extreme mortality risk and other competing events associated with ESRD overwhelm the relatively modest benefits of any specific antithrombotic therapy. This finding is consistent with other major clinical trials in the ESRD cohort, such as those involving statins, where interventions with proven benefits in the general population fail to show efficacy [32,33]. In contrast, within the DKD subgroup, treatment with cilostazol or clopidogrel was associated with unfavorable long-term survival, which may be attributed to the unique challenges of antiplatelet therapy in patients with renal dysfunction. Kidney disease itself promotes a prothrombotic state with increased platelet activation, which can blunt the effectiveness of antiplatelet medications [21]. Particularly for clopidogrel, evidence shows its antiplatelet effect is weakened in patients with CKD, leading to high platelet reactivity during treatment and potentially worse clinical outcomes [22]. While evidence for cilostazol is less direct, data from stroke prevention trials suggest that cilostazol’s efficacy may be attenuated in patients with advanced CKD [34], potentially limiting its protective effect.

Another exploratory finding in our subgroup analysis was a lower risk of LEA with cilostazol among patients with ESRD on dialysis. This observation is consistent with a previous retrospective study that found a non-significant reduction in amputation rates among dialysis patients who used cilostazol [35]. Two potential mechanisms may explain this benefit. First, in dialysis patients, adjunctive cilostazol has been shown to provide superior platelet inhibition compared to clopidogrel alone [36]. Second, cilostazol’s distinct ability to induce peripheral arterial dilation offers a direct mechanism for improving blood flow to the limbs, which could plausibly lead to better amputation outcomes compared to agents that rely on antiplatelet effects alone. However, this observation should be interpreted cautiously due to the limited number of events. Crucially, our formal interaction testing did not support a divergent treatment effect across renal strata; therefore, we treat this as a hypothesis-generating finding pending further confirmation.

This study has several limitations that should be taken into consideration. First, as a retrospective analysis utilizing a nationwide claims database, our study is subject to the inherent constraints of such data. These include the potential for misclassification bias from inaccurate diagnostic codes and the presence of unmeasured confounding variables. Specifically, the database lacks detailed clinical information such as the severity of PAD, including occlusion percentage, symptoms, and walking distance, medication dosage, and patient drug adherence. Second, non-random antiplatelet selection may introduce confounding by indication; although IPTW balanced measured covariates, residual confounding from unmeasured clinical severity and prescribing considerations may persist. Third, our study design did not capture major bleeding events, which represents a major limitation. The fundamental principle of antiplatelet therapy is balancing ischemic prevention against hemorrhagic risk. Without corresponding data on gastrointestinal or intracranial bleeding rates, we cannot comprehensively assess the comparative safety of aspirin, clopidogrel, and cilostazol. Consequently, the observed differences in all-cause mortality cannot be fully contextualized. Despite these limitations, this analysis of a large, nationwide database provides valuable real-world evidence regarding clinical prognoses in this patient population.

## Conclusion

In this observational cohort analysis, aspirin use was associated with comparable rates of LEA and MACE relative to clopidogrel and cilostazol, and a lower incidence of MALE compared to cilostazol. While aspirin was also associated with favorable long-term survival, particularly in the DKD subgroup, these retrospective findings are strictly hypothesis-generating and likely influenced by residual confounding. Given its low cost and widespread availability, aspirin remains a reasonable and practical antiplatelet option for secondary prevention in this high-risk population. However, definitive comparative effectiveness cannot be established from these observational data, and dedicated randomized controlled trials are essential to determine the optimal targeted therapy.

## Supplementary Material

Supplementary Appendix.doc

## Data Availability

The data that supports the findings of this study are available from the corresponding author upon reasonable request. Restrictions apply to the availability of these data, which were used under license for this study. Data are available from the authors only with the official permission of the Ministry of Health and Welfare, Taiwan.
