# Supplementary material for: Prognostic implications of single antiplatelet therapy in individuals developing diabetic foot disease with concurrent peripheral arterial disease
Source: Ann Med. 2026 Apr 14;58(1):2653300. doi: 10.1080/07853890.2026.2653300 (PMC13084838; doi:10.1080/07853890.2026.2653300)
Supplement: Supplementary Appendix.doc [file IANN_A_2653300_SM9151.doc]

**Appendix 1—Definition of foot diseases and comorbidities**

**Foot cellulitis and abscess:**

ICD-10 codes of L02415, L02416, L02419, L02425, L02426, L02429, L02435, L02436, L02439, L02611, L02612, L02619, L02621, L02622, L02629, L02631, L02632, L02639, L03031, L03032, L03039, L03041, L03042, L03049, L03115, L03116, L03119, L03125, L03126, L03129.

**Osteomyelitis:**

ICD-10 codes of M86061, M86062, M86069, M86161, M86162, M86169, M86261, M86262, M86269, M86071, M86072, M86079, M86171, M86172, M86179, M86271, M86272, M86279, M86361, M86362, M86369, M86461, M86462, M86469, M86561, M86562, M86569, M86661, M86662, M86669, M868X6, M86371, M86372, M86379, M86471, M86472, M86479, M86571, M86572, M86579, M86671, M86672, M86679, M868X7, M869.

**Necrotizing fasciitis:**

ICD-10 codes of M726, A480

**Gangrene:**

ICD-9 codes of 785.4, 440.24; ICD-10 codes of I96, I70261, I70262, I70263, I70268, I70269, E0852, E0952, E1152, E1352

**Peripheral arterial disease:**

ICD-10 codes of E0851, E0859, E0951, E0959, E1151, E1159, E1351, E1359, I70201, I70202, I70203, I70208, I70209, I70211, I70212, I70213, I70218, I70219, I70221, I70222, I70223, I70228, I70229,I7092, I70231, I70232, I70233, I70234, I70235, I70238, I70239, I70241, I70242, I70243, I70244, I70245, I70248, I70249, I70261, I70262, I70263, I70268, I70269, I70291, I70292, I70293, I70298, I70299, I75011, I75012, I75013, I75019, I75021, I75022, I75023, I75029, I7025, I70209, I70301, I70302, I70303, I70308, I70309, I70311, I70312, I70313, I70318, I70319, I70321, I70322, I70323, I70328, I70329, I70331, I70332, I70333, I70334, I70335, I70338, I70339, I70341, I70342, I70343, I70344, I70345, I70348, I70349, I7035, I70361, I70362, I70363, I70368, I70369, I70391, I70392, I70393, I70398, I70399, I703601, I703602, I703603, I703608, I703609, I703611, I703612, I791, I798, I7389, I739, I743, I744.

**Foot ulcers:**

ICD-10 codes of E11621, L97901, L97902, L97903, L97904, L97909, L97911, L97912, L97913, L97914, L97919, L97921, L97922, L97923, L97924, L97929, L97101, L97102, L97103, L97104, L97109, L97111, L97112, L97113, L97114, L97119, L97121, L97122, L97123, L97124, L97129, L97201, L97202, L97203, L97204, L97209, L97211, L97212, L97213, L97214, L97219, L97221, L97222, L97223, L97224, L97229, L97301, L97302, L97303, L97304, L97309, L97311, L97312, L97313, L97314, L97319, L97321, L97322, L97323, L97324, L97329, L97401, L97402, L97403, L97404, L97409, L97411, L97412, L97413, L97414, L97419, L97421, L97422, L97423, L97424, L97429, L97501, L97502, L97503, L97504, L97509, L97511, L97512, L97513, L97514, L97519, L97521, L97522, L97523, L97524, L97529, L97801, L97802, L97803, L97804, L97809, L97811, L97812, L97813, L97814, L97819, L97821, L97822, L97823, L97824, L97829, S91301A, S91302A, S91309A, S91311A, S91312A, S91319A, S91331A, S91332A, S91339A, S91351A, S91352A, S91359A, S91321A, S91322A, S91329A, S91341A, S91342A, S91349A, S91101A, S91102A, S91103A, S91104A, S91105A, S91106A, S91109A, S91111A, S91112A, S91113A, S91114A, S91115A, S91116A, S91119A, S91131A, S91132A, S91133A, S91134A, S91135A, S91136A, S91139A, S91151A, S91152A, S91153A, S91154A, S91155A, S91156A, S91159A, S91201A, S91202A, S91203A, S91204A, S91205A, S91206A, S91209A, S91211A, S91212A, S91213A, S91214A, S91215A, S91216A, S91219A, S91231A, S91232A, S91233A, S91234A, S91235A, S91236A, S91239A, S91251A, S91252A, S91253A, S91254A, S912155A, S91256A, S91259A, S91121A, S91122A, S91123A, S91124A, S91125A, S91126A, S91129A, S91141A, S91142A, S91143A, S91144A, S91145A, S91146A, S91149A, S91221A, S91222A, S91223A, S91224A, S91225A, S91226A, S91229A, S91241A, S91242A, S91243A, S91244A, S91245A, S91246A, S91249A, S91109A, S91209A.

**Lower extremity amputation (LEA):**

***Minor-LEA***

ICD-10 diagnostic codes of Z899, Z89411, Z89412, Z89419, Z89421, Z89422, Z89429, Z89431, Z89432, Z89439, Z89441, Z89442, Z89449.

ICD-10 procedure codes of 0Y6M0Z0, 0Y6M0Z4, 0Y6M0Z5, 0Y6M0Z6, 0Y6M0Z7, 0Y6M0Z8, 0Y6M0Z09, 0Y6M0ZB, 0Y6M0ZC, 0Y6M0ZD, 0Y6M0ZF, 0Y6N0Z0, 0Y6N0Z4, 0Y6N0Z5, 0Y6N0Z6, 0Y6N0Z7, 0Y6N0Z8, 0Y6N0Z09, 0Y6N0ZB, 0Y6N0ZC, 0Y6N0ZD, 0Y6N0ZF, 0Y6P0Z0, 0Y6P0Z1, 0Y6P0Z2, 0Y6P0Z3, 0Y6Q0Z0, 0Y6Q0Z1, 0Y6Q0Z2, 0Y6Q0Z3, 0Y6R0Z0, 0Y6R0Z1, 0Y6R0Z2, 0Y6R0Z3, 0Y6S0Z0, 0Y6S0Z1, 0Y6S0Z2, 0Y6S0Z3, 0Y6T0Z0, 0Y6T0Z1, 0Y6T0Z2, 0Y6T0Z3, 0Y6U0Z0, 0Y6U0Z1, 0Y6U0Z2, 0Y6U0Z3, 0Y6V0Z0, 0Y6V0Z1, 0Y6V0Z2, 0Y6V0Z3, 0Y6W0Z0, 0Y6W0Z1, 0Y6W0Z2, 0Y6W0Z3, 0Y6X0Z0, 0Y6X0Z1, 0Y6X0Z2, 0Y6X0Z3, 0Y6Y0Z0, 0Y6Y0Z1, 0Y6Y0Z2, 0Y6Y0Z3.

***Major-LEA***

ICD-10 diagnostic codes of Z89511, Z89512, Z89519, Z89611, Z89612, Z89619, Z89621, Z89622, Z89629.

ICD-10 procedure coeds of 0Y6C0Z1, 0Y6C0Z2, 0Y6C0Z3, 0Y6D0Z1, 0Y6D0Z2, 0Y6D0Z3, 0Y6H0Z1, 0Y6H0Z2, 0Y6H0Z3, 0Y6J0Z1, 0Y6J0Z2, 0Y6J0Z3.

**Hypertension:**

ICD-10 codes of I10, I110, I120, I129, I130, I1311, I132, I150, I151, I152, I158, I159, I160, I161, I169.

**Dyslipidemia:**

ICD-10 codes of E780, E781, E782, E783, E784, E785.

**Coronary heart diseases:**

ICD-10 codes of I220, I221, I222, I228, I229, I230, I231, I232, I233, I234, I235, I236, I237, I238, I2101, I2102, I2109, I2111 I2119 I2121, I2129, I213, I214, I200, I240, I248, I249, I252, I2510, I25110, I25111, I25118, I25119, I255, I256, I25700, I25701, I25708, I25709, I25710, I25711, I25718, I25719, I25720, I25721, I25728, I25729, I25730, I25731, I25738, I25739, I25750, I25751, I25758, I25759, I25760, I25761, I25768, I25769, I25790, I25791, I25798, I25799, I25810, I25811, I25812, I2582, I2583, I2584, I2589, I255, I256, I2589, I259.

**Cerebral vascular accident:**

***Ischemic stroke***

ICD-10 codes of I63, I672, I6781, I6782, I6789, I679, I688, G450, G451, G452, G458, G459.

***Hemorrhagic stroke***

ICD-10 codes of I60, I61, I62.

**Diabetic kideny disease:**

ICD-10 codes of E1121, E1122, E1129, E1321, E1322, E1329, E1165, N181, N182, N183, N184, N185, N186, N189, N19, Z940, Z4931, Z4901, Z4902, I129, I120, I1310, I130, I1311, I132

**End-stage renal disease:**

ICD-10 codes of N185, N186, N19, Z992, Z4931, Z4901, Z4902, I120, I1311, I132.

**Appendix 2— Brief summary of related procedure payment codes of NHIRD**

| **Code** | **Procedure item** | **Code** | **Procedure item** |
| --- | --- | --- | --- |
| 33066B | Tomography (each) | 68048B | Percutaneous transluminal septal myocardial ablation |
| 33070B | computered tomography (C. T.)-without contrast | 68049B | Thoracoscopic Pericardial Window |
| 33071B | computered tomography ( C. T.)-with contrast | 68050B | Arrhythmia surgery via atriotomy |
| 33072B | computered tomography ( C. T.)- with/without contrast | 68051B | Ventricular Assist System implantation |
| 33073B | Pulmonary angiography | 68052B | Cardiopulmonary bypass |
| 33074B | P.T.A. (percutaneous transluminal angioplasty): simple | 69001B | Embolectomy,arterial |
| 33075B | T.A.E.(trans-arterial embolization) | 69002B | Embolectomy,arterial catheter |
| 33076B | Percutaneous coronary dilatation-one vessel | 69003B | Thrombectomy, venous |
| 33103B | CT Guide biopsy | 69004B | Arterial endarterectomy with or without bypass graft |
| 33104B | Direct pulmonary venography | 69005B | Exploration, vascular |
| 33092B | Fluoroscopic reduction of intussusception | 69019B | Stripping, subfascial, radical as Linton type with or without skin graft |
| 33093B | Bilateral reno-venous sampling | 69020B | Ligation and division of short saphenous vein at sapheno- popliteal junction |
| 33095B | Revision of percutaneous nephrostomy tube(Pigtail) | 69021C | Suture, ligation or stripping of minor varicose vein |
| 33096B | Swallowing video fluorography | 69022B | Embolectomy, pulmonary artery |
| 33097B | IV-DSA | 69023B | Excision and graft bypass or direct repair of A-V fistula, neck or extremity |
| 33113B | Transjugular intrahepatic portosystemic shunt | 69035B | Bental procedure |
| 33114B | Transcatheterclosureofpatentductusarteriosus>=2.5mm | 70405B | Repair of diaphragmatic hernia trans-abdominal |
| 33115B | P.T.A. (percutaneous transluminal angioplasty): complex | 69036B | Excision and graft bypass or direct repair A-V fistula of chest or abdomen |

Please check the link below: https://data.gov.tw/dataset/9405 for complete coding book (in Chinese characters).
